# Broadband Transient Fluorescence Reveals a Two-State Picture of Peridinin

**DOI:** 10.1021/acs.jpclett.6c02559

**Published:** 2026-10-14

**Authors:** Alina Bach, Constantin Jaschke, Heyuan Liu, Hailong Chen, Erika Keil, Eckhard Hofmann, Yuxiang Weng, Jan P. Götze, Benjamin P. Fingerhut, Jürgen Hauer

**Affiliations:** ○ Department of Chemistry, Professorship of Dynamic Spectroscopy, TUM School of Natural Sciences, 9184 Technical University of Munich, 85748 Garching, Germany; ‡ Department of Chemistry and Centre for NanoScience, 9183 Ludwig-Maximilians-Universität München, 81377 München, Germany; § Laboratory of Soft Matter Physics, Beijing National Laboratory for Condensed Matter Physics, 47849 Institute of Physics, Chinese Academy of Sciences, Beijing 100190, China; ∥ School of Physical Science, University of the Chinese Academy of Sciences, Beijing 100049, China; ⊥ Faculty of Mathematics and Physics, Charles University, 113 36 Prague, Czech Republic; # Biophysics, Department of Biology and Biotechnology, Ruhr-University Bochum, 44780 Bochum, Germany; ∇ Institut für Chemie und Biochemie, 54203 Freie Universität Berlin, 14195 Berlin, Germany

## Abstract

Peridinin is a light-harvesting apocarotenoid whose excited-state relaxation is often described by population transfer among 1B_u_, 2A_g_, and intramolecular charge-transfer states. Here broadband-detected transient fluorescence reveals a simpler picture for peridinin in ethylene glycol. Following optical excitation, we observe instantaneous blue-green emission from the bright 1B_u_ state that depopulates with a 150 fs time constant and directly gives rise to weak red emission from the lowest 2A_g_ state, which decays in 12 ps. The presence of an isosbestic point and the absence of additional fluorescence components indicate direct internal conversion without a resolvable hot S_1_ or a separate equilibration process involving a charge-transfer state. XMS-RASPT2 and DFT/MRCI calculations reproduce this behavior by showing strong displacement of the 1B_u_ and 2A_g_ minima from the Franck–Condon region and weak oscillator strength at the relaxed 2A_g_ minimum. The results identify peridinin as an emitter from its 1B_u_ and 2A_g_ states and revise the interpretation of its charge-transfer-like signatures.

## Graphical abstract

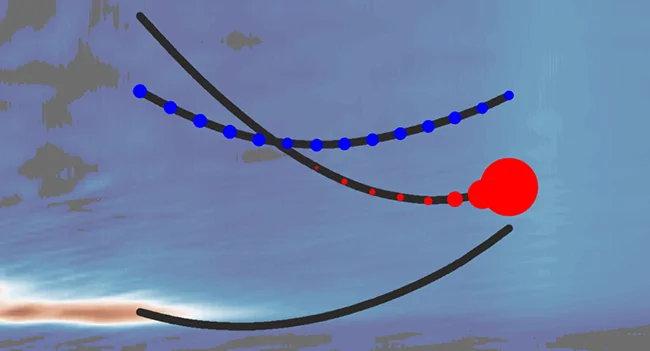

Carotenoids are a central class of chromophores in photosynthetic systems, serving as accessory pigments to and photoprotectors of chlorophylls.^1^ The spectroscopic properties of carotenoids are defined by their polyenic backbone.^2,3^ Assuming idealized *C*_2*h*_ geometry, the electronic states of carotenoids are of either A_g_ or B_u_ character. As the symmetry of an orbital needs to change for a spectroscopically allowed transition, A_g_ to B_u_ (A_g_) transitions are strongly allowed (forbidden). Although the validity of this assumption has been disputed,^4^ there is consensus that the initially excited state in carotenoids is not necessarily the one with the overall lowest energy. In the photophysically relevant Franck–Condon (FC) region,^5^ the second excited singlet state is typically assigned to the optically accessible state with B_u_ character.^6^ It exhibits an ultrashort lifetime that depends strongly on the polyene chain length and on solvent polarity. For β-carotene with 11 conjugated double bonds, the lifetime of the initially excited state changes from 180 to 130 fs in hexane and CS_2_, respectively.^7^ The state of 1B_u_ character relaxes via ultrafast internal conversion to the final excited state with 2A_g_ character, carrying the excited-state minimum.^8–10^ The experimentally observed transfer time decreases with increasing chain length.^11^ This has strong implications for the emissive properties of the molecules: even for high symmetry carotenoids, it is possible to observe (weak) emission from the 1B_u_-character state (>9 double bonds), the 2A_g_-character state (<9 double bonds), or both (8–9 double bonds).^12,13^

If the *C*_2*h*_ geometry of the carotenoid is lowered by substitution of the polyene backbone, the selection rules become less restrictive, as is the case for peridinin (see structure in Figure 1). Peridinin is the main light-harvesting pigment of the peridinin–chlorophyll *a* binding protein complex (PCP) found in dinoflagellates and shows a highly unusual chemical structure. It features an allene moiety, terminal carbonyls, and a lactone group fused with the polyenic backbone. This lactone group has a strong electron-pulling effect, and was suggested as the structural basis for an intramolecular charge transfer (ICT) state.^14,15^ There is an ongoing debate whether the 2A_g_ state or an ICT state is the main energy donor to Chl *a* in PCP.^16,17^ Even for peridinin in solution, the nature of the ICT state and its relation to the other excited states remain disputed.^18^ To give a coarse overview, the ICT state was interpreted as a distinct electronic state with a large dipole moment that may quench the S_1_ state.^15,19^ Alternatively, ICT and S_1_ were suggested to mix coherently to form a new excited-state landscape in polar solvents.^20^ In another interpretation, the ICT state was seen as the result of structural distortion after photo excitation, accompanied by redistribution of π-electron density.^18,21^

**Figure 1:**
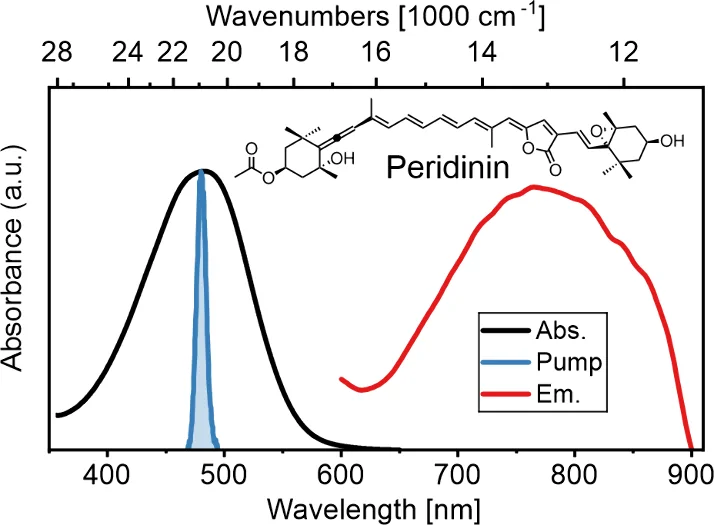
Absorption (black) and emission (red) spectra of peridinin (see molecular structure) dissolved in ethylene glycol. The emission spectrum was recorded at an excitation wavelength of 480 nm. The filled blue curve shows the spectrum of the excitation pulse used in the time-resolved fluorescence experiment.

The main experimental method used to study the ICT state and peridinin’s photodynamics in general is transient absorption (TA). Peridinin’s TA signal at early delay times is dominated by ground-state bleach and 1B_u_-stimulated emission.^22^ Excited-state absorption from the initially excited state is weak in the visible and peaks in the near-infrared spectral range.^22^ The lowest-lying excited state is populated after internal conversion, commonly ascribed to relaxation from a vibrationally hot state (“hot S_1_”).^23,24^ Relaxation toward the excited-state minimum occurs on a subpicosecond time scale, causing the strong excited-state absorption signal to continuously blueshift and narrow. Such a gradual spectral evolution is problematic for global target analysis,^25^ as the signatures of the retrieved spectral species are not sufficiently separated in their time and frequency dependence.^26^ This makes it difficult to define the number of spectral species necessary for a global fit to the TA data and leads to the interpretational ambiguities sketched above.

Ultrafast fluorescence-detected methods are highly promising for unraveling the convoluted relaxation dynamics in carotenoids, especially compared to TA studies, as transient fluorescence signals are devoid of the excited-state absorption signatures that often dominate TA signals. The static fluorescence signal of peridinin is shown in Figure 1 as a red line. The emission stems from the nominally dark 2A_g_ state.^27^ Stimulated emission from the ICT state was reported to peak around 950 nm.^20^

Ultrafast fluorescence up-conversion experiments carried out on peridinin in methanol reported excited-state lifetimes of 130 fs^28^ and 192 fs,^29^ detected at a single emission wavelength. The lifetime of the relaxed lowest excited state is—unlike in linear, unsubstituted carotenoids—also strongly solvent-dependent, with values of 10 and 156 ps in methanol and *n*-hexane, respectively. Even though broadband methods that are in principle capable of the simultaneous detection of 1B_u_ and 2A_g_ emission have been developed,^30–33^ they have not been applied to peridinin yet.

In this work, we aim to apply broadband-detected transient fluorescence to peridinin in ethylene glycol solution to gain a new perspective on the molecule’s ultrafast deactivation network. Specifically, we want to distinguish the conventional three-state energy-level model of peridinin (1B_u_, 2A_g_, ICT) from a two-state model (1B_u_, 2A_g_) in which the geometries at the FC point and at the excited-state minimum exhibit large displacements along an internal coordinate.^18,34^ In the latter, the spectral signatures interpreted as the ICT state are not due to a separate electronic state but result from structural relaxation followed by solvent reorganization dynamics.

## Sample Preparation

Peridinin was extracted from *Amphidinium carterae* membranes with 2-butanol. The organic phase was dried and redissolved in 30% v/v methanol/30% v/v acetonitrile, applied to a C18-Flash column (Chromabond Flash RS4 4g; Macherey-Nagel, Germany), and eluted with a gradient to 100% v/v acetonitrile. Peak fractions were dried, resuspended in methanol, and subjected to HPLC purification, following the protocol of Jeffrey et al.^35^ Briefly, the prepurified peridinin was applied to a C18 column (ZORBAX SB-C18 4.6 mm × 250 mm, 5 μm; Agilent, Germany) equilibrated with methanol/100 mM ammoniumacetate, pH 7.2. Pure peridinin was eluted with a gradient to 90% v/v acetonitrile followed by a gradient to 18% v/v acetonitrile/80% v/v ethyl acetate. Peak fractions were dried and resuspended in ethanol. After spectral quality control, aliquots were dried and stored at 203 K. For measurements, aliquots were resuspended in ethylene glycol. For the time-resolved fluorescence measurements detailed below, the peridinin solution in ethylene glycol was placed in a closed-loop circulation system to avoid photodegradation during prolonged laser irradiation. The sample was circulated through a 1 mm path-length quartz cuvette (by Yixing Purshee Optical Elements Co., Ltd.) connected to flexible tubing on both sides driven by a peristaltic pump (model: KZQ1-BQ50-1J, Longer Precision Pump Co., Ltd., China). The optical density at the central wavelength of the excitation pulse (see Figure 1) was set to OD = 0.2. The fluorescence excitation pulse was focused onto the flowing sample inside the cuvette, ensuring that the irradiated volume was continuously refreshed throughout the measurement.

## Spectroscopic Methods

The absorption and emission spectra depicted in Figure 1 were recorded in a PerkinElmer 365 spectrometer and an Edinburgh FS5 spectrometer, respectively. We employed fluorescence noncollinear optical parametric amplification spectroscopy (FNOPAS) to detect the time-resolved emission of peridinin in ethylene glycol. The method was described in detail previously^33,36–38^ and successfully applied to photosynthetic pigments and pigment–protein complexes.^39,40^ Briefly, the output of a Ti:sapphire amplified laser system (Solstice, Spectra-Physics, 800 nm, 100 fs, 2 kHz) was divided in two beams of equal intensity. One beam was frequency-doubled in a 2 mm β-barium borate (BBO) crystal, serving as the pump pulse in FNOPAS. The other fraction of the fundamental laser beam was utilized to pump an optical parametric amplifier (TOPAS, Spectra-Physics, see the blue line in Figure 1 for the excitation spectrum, 0.15 μJ at the sample position). The fluorescence triggered by the OPA output in the sample was focused into a 1 mm thick BBO crystal (θ = 31.5°, φ = 0°) and amplified by the 400 nm pump pulse. The amplified fluorescence and the strong superfluorescence ring from the NOPA process were recorded with a custom-built fiber-coupled spectrometer equipped with a polychromator (Princeton Instruments, Acton SpectraPro SP-2150) and a high-speed CMOS detector (trSpectra, Nanjing, China). The superfluorescence background was subtracted by blocking and unblocking the OPA arm with a synchronized mechanical chopper at 625 Hz. The nonuniform gain curve of the NOPA-BBO crystal was corrected for in postprocessing, following previously published procedures.^40^

## Computational Methods

The theoretical approach follows the work of Zhang et al.^10^ Simulations were performed for a full-chromophore model of peridinin discussed previously.^18,41^ Excited-state geometries, energies, and properties were computed at the extended multistate restricted active space second-order perturbation theory (XMS-RASPT2) level of theory with the OpenMolcas v23.06 program.^42–44^ An active space of 14 electrons in 14 orbitals was employed, among which a subspace of four electrons in the five closest-to-frontier orbitals was treated at the full configuration interaction level of theory (RAS2 excitation space). Excitations between RAS2 and the formally occupied and virtual orbital subspaces (RAS1 and RAS3 excitation spaces, respectively) were restricted to single and double excitations (RASSCF­(14/14, 2:4/5:2)). State averaging considered the four lowest eigenstates. The RASSCF approach was tailored to recover the accuracy of a complete active space self-consistent field (14/14) treatment with 0.01 eV accuracy while conserving the state ordering of the 2A_g_- and 1B_u_-like reference states. The 2A_g_-like reference was used for self-consistent implicit solvation modeling with the conductor-like polarizable continuum model (CPCM), employing nonequilibrium conditions and a dielectric constant of 37 for ethylene glycol.^45^ The ANO-L-VTZP basis set was used as the excitation energies displayed slow basis set convergence.^46^ For the perturbative correction, the IPEA shift was switched off, and the advanced σ^2^ regularization approach was employed to ensure the removal of intruder states at marginal distortion of excitation energies.^47^ The collective nuclear displacement coordinate was generated by principal component analysis (PCA) of the XMS-RASPT2-optimized 1A_g_, 1B_u_, and 2A_g_ minimum geometries using the lowest PCA with 95% explained variance (see the Supporting Information (SI)) and discretized to 14 single-point evaluations.^48^ The lowest PCA coordinate reflects the bond-order inversion upon relaxation from the FC geometry to the conformational region containing the excited-state minima. This leads us to the relative mean bond length (RMBL) metric, which some of us have employed previously to describe excited-state relaxation in β-carotene.^10^ Oscillator strengths were calculated via transition dipole moments in length gauge. Additionally, oscillator strengths were computed with the multireference configuration interaction approach based on density functional theory/multireference configuration interaction (DFT/MRCI)^49,50^ using the 2022 Hamiltonian interfaced to ORCA (version 6).^51,52^ The required orbital basis was obtained from a BHLYP/def2-SVP calculation,^53,54^ using a 10^–7^ energy convergence threshold and the CPCM implicit solvent model for acetonitrile (for which the dielectric constant is similar to that of ethylene glycol).^45^ The subsequent DFT/MRCI calculation computed the three lowest singlet states (i.e., the ground state, 1A_g_, and two excited states, 1B_u_ and 2A_g_), with transitions starting from any of those states; more states were not considered, as test calculations did not indicate any additional states to become as low in energy at any point. We focus here on the energies of the excited states, along the RMBL coordinate, as well as the oscillator strengths (*f*_*x*→0_) for spontaneous emission from each excited state. The corresponding datasets can be found in the SI; they are in good agreement with the XMS-RASPT2 results shown in the main article.

## Experiment

Figure 2a shows the transient fluorescence signal of peridinin in ethylene glycol solution, dispersed in detection wavelength, and as a function of time delay between the fluorescence and the FNOPAS pump pulse. Between 520 nm—the blue edge of our detection window—and 700 nm, we observe a strong, short-lived signal whose rise cannot be resolved with the instrument’s 100 fs time resolution (see the SI for cuts along selected detection wavelengths). Centered at 735 nm, we observe a low-amplitude spectral feature, highlighted by a white dashed box in Figure 2a and shown as a zoom-in in Figure 2b. We can resolve an ultrafast rise and a much slower decay. The signal amplitude is close to zero at the end of the 10 ps window of our experiment. Global analysis^25^ of the dataset reveals that only two spectral species are required for a convergent fit. Figure 2c shows the evolution-associated spectra (EAS), assuming a sequential decay from the initial component (150 fs lifetime) to the second component (11.9 ps), and back to the electronic ground state. The shape and decay time of the first component allow us to assign this feature to fluorescence from the initially excited state. The next EAS component—the blue line in Figure 2c—rises with the decay time of the initial component and then decays back to the ground state with a time constant of 11.9 ps. Its spectrum agrees well with the shape of the time-integrated fluorescence spectrum (dashed line). This allows us to assign the second EAS to the lowest-lying excited state of peridinin. To further support the finding that two kinetic species suffice to describe the data, i.e., that the fit requires no intermediate species between the initially excited and the lowest-lying excited state, we show cuts along the spectral axis at delay times indicated in the inset of Figure 2c. The dotted vertical line highlights the isosbestic point, indicating a direct transfer between the green and red-emitting species. This means that the data are reproduced by a fit that does not require an additional “hot S_1_ component”, usually found in TA experiments on a time scale of hundreds of femtoseconds.^23^

**Figure 2:**
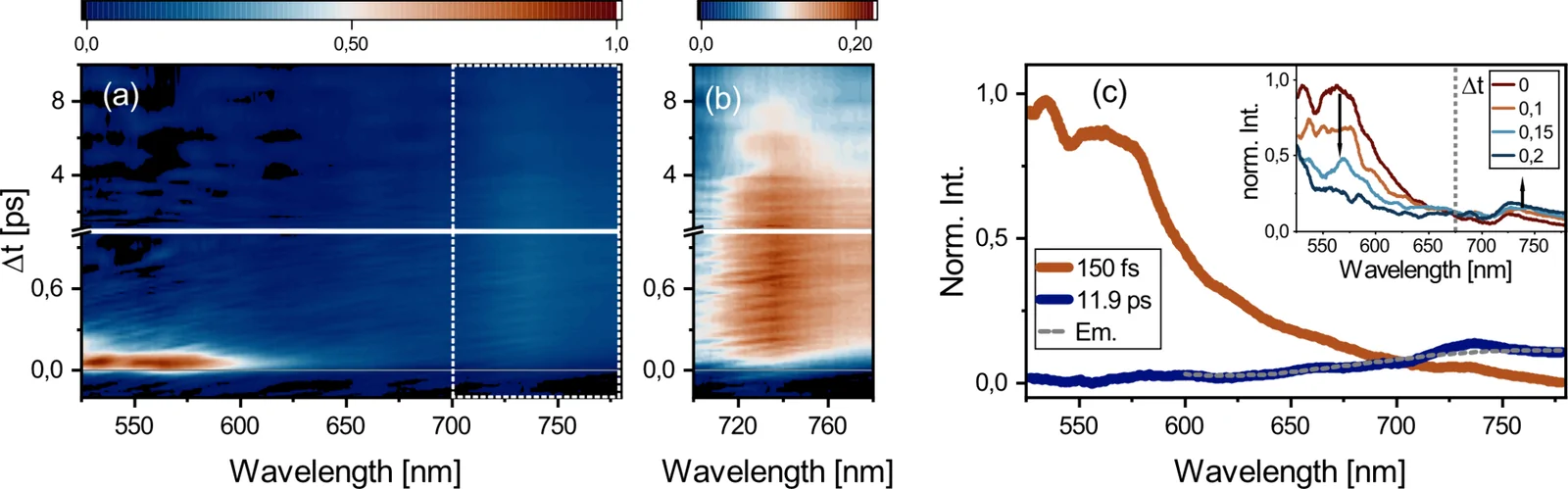
(a) Detection-wavelength-resolved transient fluorescence spectrum of peridinin dissolved in ethylene glycol. (b) Zoom-in of the signal marked by the white box (dashed line) in (a). (c) Evolution-associated spectra extracted from the measured data in (a). The labels indicate species lifetimes. The inset shows cuts at the indicated delay times in picoseconds, with an isosbestic point at 675 nm (dotted vertical line). The dashed gray line shows the fluorescence spectrum taken from Figure 1.

## Theory

The observed fluorescence properties of peridinin are analyzed within an XMS-RASPT2 model that involves the 1A_g_ ground state and the two excited states 1B_u_ and 2A_g_ (Figure 3). Compared to previous work,^18,34,41,55^ we find that at the FC geometry the 1B_u_ state is the only optically accessible state and intersects with the second excited state of 2A_g_ character (RMBL coordinate ∼ 1.025). Diabatic state labels (1B_u_ and 2A_g_) at each point were assigned based on the dipole moments of the states (not shown). At high RMBL values, the first excited state shows 1B_u_ character. After the intersection, the 1B_u_ state is the second excited state. Therefore, we assigned 1B_u_ character to be the lowest excited state near the FC point (at large RMBL values) and the second excited state after the intersection, and vice versa for 2A_g_. The observed state ordering is a direct consequence of the high-level XMS-RASPT2 treatment with the large ANO-L-VTZP basis set and an improved ground-state geometry optimized at the XMS-RASPT2 level of theory.

**Figure 3:**
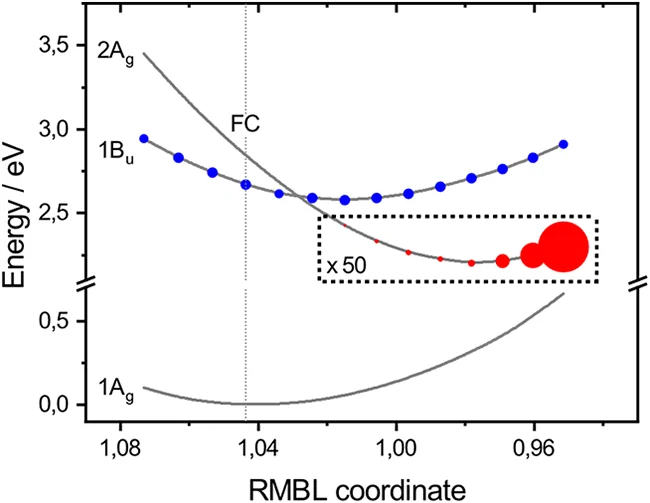
Calculated XMS-RASPT2 state energies (lines) and oscillator strengths (dots) connecting the two lowest electronically excited states of peridinin to the electronic ground state. The dot size is normalized to the maximal oscillator strength from the 1B_u_ state (blue); the oscillator strength from the minimum of the 2A_g_ state is scaled by a factor of 50 for visual comparison.

The oscillator strength, calculated at each point along the potential energy surfaces, is used for comparison with the data in Figure 2. We plot the XMS-RASPT2-computed oscillator strength in Figure 3 (blue and red dots) in addition to the corresponding state energies along the RMBL displacement coordinate. The switch in the static dipole of each state is also reflected in a change in the oscillator strengths: At high RMBL values (Figure 3, left), the first excited state with 1B_u_ character carries all the oscillator strength (blue dots); the 2A_g_ state is optically dark in the FC region. Only at the 2A_g_ minimum is the corresponding oscillator strength non-negligible (red dots). This is a result of the mixing of wave function coefficients of the 1A_g_, 2A_g_ and 1B_u_ states in the RAS and XMS wave function (red dots; see the SI for CSF and XMS-CASPT2 mixing coefficients).

We start the discussion by ascribing the two kinetic species shown in Figure 2c to their molecular origins with the aid of theory. Subsequently, we discuss our findings in the light of previous TA studies. We then turn to a general discussion of relaxation kinetics in peridinin and present new insights derived from our data and modeling.

The initial component with a 150 fs lifetime clearly stems from the optically bright 1B_u_ state as detailed above. The green emission we observe, see 150 fs EAS in Figure 2c, corresponds to the computed 1B_u_–1A_g_ energy gap (Figure 3), supporting this interpretation. The lifetime of the 1B_u_ state in carotenoids is limited by internal conversion to the lower-lying 2A_g_ state.^6^ Accordingly, we find a conical intersection between the 2A_g_ and 1B_u_ surfaces in our two-state model of peridinin (see Figure 3). This non-adiabatic curve crossing typically leads to fluorescence quenching, as 2A_g_ is completely dark (nonfluorescent) in unsubstituted carotenoids. Peridinin, in contrast, retains a small oscillator strength in the 2A_g_ state, as evidenced by the red circles in Figure 3. We attribute this weak emission to 2A_g_–1A_g_–1B_u_ mixing arising from symmetry-breaking in peridinin (see Figure S4 for details). Hence, we attribute the weakly red-emitting species in Figure 2c to the 2A_g_ state.

A main experimental finding of this work is that only two kinetic components suffice to describe the transient fluorescence dataset, as supported by the observation of an isosbestic point at 675 nm, see inset of Figure 2c. The situation is much more complicated in TA spectroscopy of peridinin in polar solvents. In general agreement with the literature,^22,56^ Niedzwiedzki et al.^22^ found four spectral components in various polar solvents and ascribed them to S_2_ (1B_u_), hot S_1_, further structural relaxation on S_1_ (2A_g_) or S_1_ → ICT transfer^20,56,57^ and relaxation back to S_0_ (1A_g_). While we also observe S_2_ decay as the first component, the relaxation of a vibrationally hot S_1_ on a time scale of several hundred femtoseconds is missing from our transient fluorescence data. We note that time-resolved fluorescence detection itself can resolve vibrational cooling, as witnessed by several reports using FNOPAS^40^ or fluorescence upconversion.^58,59^ The lack of a “hot S_1_” component in the transient fluorescence data in Figure 2 is, however, readily explained by the simulations in Figure 3. For the case of peridinin, the oscillator strength (red circles in Figure 3) is negligible for the lower-lying excited state except for regions near the excited-state minimum. Hence, fluorescence from vibrationally hot levels on the 2A_g_ state is less likely to be detected than emission from the vicinity of the 2A_g_ minimum. We note that the oscillator strength increases with decreasing 1A_g_-2A_g_ energy gap; see Figure 3. This increase of signal intensity toward longer wavelengths is not observed in Figure 2b, most likely due to worsening phase matching conditions toward the edge of the detection window.^37^

Before discussing our findings further, we briefly recapitulate the current view in the literature. One popular interpretation of the relaxation dynamics of peridinin in polar solvents is that—after ultrafast relaxation of the initially excited 1B_u_ state—the population branches into hot S_1_ and the ICT state.^22,56^ Subsequently, the relaxed S_1_ and ICT may exchange population in an equilibration step.^20^ It is remarkable that—within this model—the S_2_ → hot S_1_ transfer can be resolved, whereas the S_2_ → ICT step is faster than the instrument response function, even in experiments with sub-10 fs excitation pulses in the visible.^60^ However, the stimulated emission band in the NIR associated with the ICT state shows a solvent-dependent rise time in the sub-ps regime.^20^ With our revised model of the electronic structure of peridinin, we can explain these seemingly controversial findings. For relaxation along the RMBL coordinate, the 2A_g_ minimum is clearly displaced from the FC region (Figure 3). Additionally, the energy difference between the FC geometry and the 2A_g_ minimum geometry is relatively large, indicating a substantial reorganization process. We arrive at the following revised interpretation: optical excitation populates the lowest excited state (1B_u_) in the FC region. The majority of the excited-state population relaxes to the 2A_g_ state via internal conversion. This scenario—prior to solvent relaxation—is fully captured by Figure 3. Solvent relaxation (which is beyond the calculations employed in this work) will affect the 1B_u_ state more strongly,^61^ causing its energy to sink below the 2A_g_ minimum. Hence, we propose that the quenching of the 2A_g_ lifetime to 11.9 ps occurs not via an independent ICT state^15^ but rather via the 1B_u_ state after solvent relaxation (see the SI for a sketch of the potential energy surfaces). This mechanism explains the 650 fs rise of the ICT stimulated emission reported for ethylene glycol by Zigmantas et al.,^20^ as such a subpicosecond component matches well with solvent response times in literature.^62^ As reported repeatedly,^20,22^ the lifetime of the ICT-related features and the 2A_g_ state is similar, regardless of solvent, temperature, and detection range. One way to reconcile this finding with our model is to consider mixing between the states, which we already introduced to explain the non-negligible 2A_g_ emission, as shown in Figure 3. Such wave function mixing effects will be stronger upon closing of the 1B_u_–2A_g_ energy gap upon solvent-induced stabilization. We speculate that such enhanced mixing renders the excited state inseparable, meaning that they decay with a common lifetime. This notion of a collective S_1_/ICT state was introduced previously by Zigmantas et al.^20^ We simplify the model by replacing the ICT state with a solvent-stabilized state of 1B_u_ character. Further studies will quantify the influence of solvent stabilization and wave function mixing in peridinin.

Another intriguing experimental finding was the lack of a discernible S_2_ → ICT transfer time prior to solvent relaxation.^60^ Our model readily explains this if we assume that the initial 1B_u_ → 2A_g_ relaxation is incomplete, i.e. that after optical excitation the 1B_u_ population will bifurcate, with a small fraction remaining in the 1B_u_ state. In future work, simulations based on such an assumption will be verified against TA data.

Our work shows that peridinin acts as a dual emitter, initially from 1B_u_ and subsequently from 2A_g_. The additional (albeit weak) 2A_g_ emission positions peridinin as a much more efficient energy donor in a photobiological context. Peridinin’s 2A_g_ state is preferably tuned to chlorophyll *a* as the acceptor pigment in the PCP complex.^63,64^ Our insights presented here indicate that the biologically relevant energy-donor role of peridinin stems from emission from the 2A_g_ state. TA signals found in PCP and attributed to peridinin’s ICT state^16,65^ indicate that the 1B_u_ stabilization described here is also relevant in a protein environment.

In summary, we present broadband-detected, time-resolved fluorescence data for peridinin dissolved in ethylene glycol. The underlying kinetics is surprisingly simple compared to TA spectroscopy results.^22^ We explain our findings using a model in which the minima of the 1B_u_ and 2A_g_ states are strongly displaced from the FC geometry. We propose that relaxation along the RMBL coordinate toward these minima and solvent-relaxation effects are the origin of the spectral signatures previously assigned to the ICT state.

## Supplementary Material



## Notes

The Supporting Information is available free of charge at https://pubs.acs.org/doi/10.1021/acs.jpclett.6c02559. Data for all figures shown in the manuscript can be downloaded under 10.5281/zenodo.23338089.

Details on global analysis results and computational methods, supporting simulation results, and sketches of potential energy surfaces before and after solvent relaxation (PDF)
