# Supplementary material for: Broadband Transient Fluorescence Reveals a Two-State Picture of Peridinin
Source: J Phys Chem Lett. 2026 Oct 14;17(43):12310–7. doi: 10.1021/acs.jpclett.6c02559 (PMC13726465; doi:10.1021/acs.jpclett.6c02559)
Supplement: Supplementary file 1 [file jz6c02559_si_001.pdf]

Supporting Information

Broadband Transient Fluorescence Reveals a Two-State Picture of Peridinin

Alina Bach[1†], Constantin Jaschke[2†], Heyuan Liu[3,4], Hailong Chen[3,4], Erika Keil[5], Eckhard Hofmann[6], Yuxiang Weng[3,4], Jan P. Götze[7], Benjamin P. Fingerhut[2*], and Jürgen Hauer[1*]

[1]Department of Chemistry, Professorship of Dynamic Spectroscopy, TUM School of Natural Sciences, Technical University of Munich, 85748 Garching, Germany

[2]Department of Chemistry and Centre for NanoScience, Ludwig-Maximilians-Universität München, München 81377, Germany

[3]The Laboratory of Soft Matter Physics, Beijing National Laboratory for Condensed Matter Physics, Institute of Physics, Chinese Academy of Sciences, Beijing 100190, China

[4]School of Physical Science, University of the Chinese Academy of Sciences, Beijing 100049, China

[5]Faculty of Mathematics and Physics, Charles University, Prague 11336, Czech Republic

[6]Biophysics, Department of Biology and Biotechnology, Ruhr-University Bochum, D-44780 Bochum, Germany

[7]Institut für Chemie und Biochemie, Freie Universität Berlin, 14195 Berlin, Germany

[†]contributed equally

[*]corresponding authors. Emails: juergen.hauer@tum.de, benjamin.fingerhut@cup.lmu.de

1. Materials and Experimental Methods

Figure S1 exemplifies the quality of the global analysis results. The model is sequential with two excited states, where only the higher lying one is optically excited at Δt = 0 fs, and a ground state. The spectral positions of the shown cuts correspond to the emission maximum of the initially excited state ($1B_u$ at 575 nm) and the lower-lying excited state ($2A_g$ at 735 nm, see Fig. 2(a) in the main text). Notably, two states suffice for a satisfactory, convergent fit. This means that contributions from a vibrationally hot $2A_g$ state are not necessary to describe transient fluorescence data.

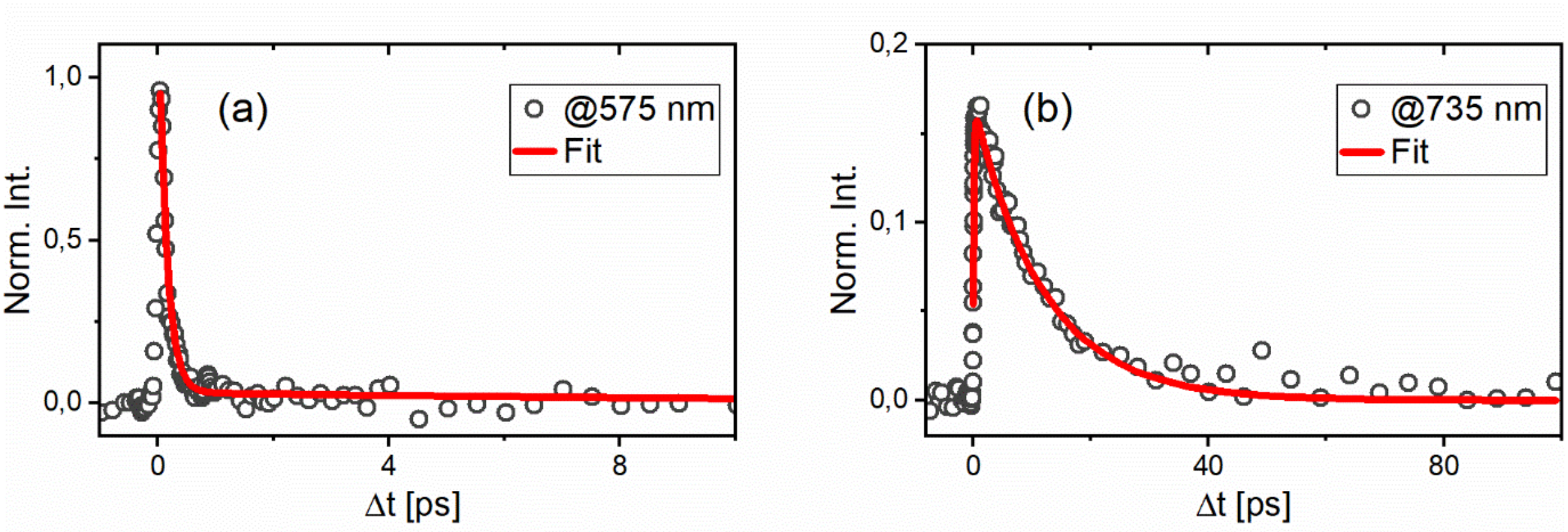

Figure S1: (a) cut through the broadband-detected time-resolved fluorescence data at the emission maximum of the $1B_u$ state at 575 nm. See Fig. 2(a) of the main text for the spectrally resolved data. The red line stems from a fit of a sequential global analysis model, based on two excited states and one ground state. The evolution-associated spectra are shown in Fig. 2(c). At 575 nm, the dominating contribution is an ultrafast decay with a 150 fs time constant. (b) Cut at 735 nm, the maximum of the $2A_g$ emission. Here, the data is described by a 150 fs rise and a 11.9 ps decay.

2. Computational Methods

In the following we provide a technical description of the employed electronic structure methods, as well as the conformer selection for ground state geometry optimization. Electronic structure calculations have been performed with OpenMolcas v23.06[1]. Unless stated otherwise, the calculations applied the default settings of the respective quantum chemistry programs. Nuclear gradients were evaluated analytically. For numerical efficiency of XMS-CASPT2 computations, simulations were performed for a full-chromophore model of peridinin (see Figure S2 and S3) already employed by Taffet *el al.*[2] and Wagner *et al.*[3] The model reduces peridinin to the chromophore by truncating at the boundary to the β-rings, replacing them with

hydrogen. DFT/MRCI simulations are used to validate the high accuracy of the structural model (see SI Sec. **3.2**).

2.1 Electronic Structure Method

Calculations on the CPCM-XMS-CASPT2 level of theory employed the ANO-L-VTZP basis set. Smaller, commonly used double-ζ basis sets for carbon atoms show a large error due to the slow basis set convergence of the $1\widetilde{B_u}$ state. Cholesky decomposition was used (threshold: 10^{-6} Hartree atomic units), and no symmetry restrictions were imposed.

The wavefunction for the multi-state perturbation theory treatment was obtained from an equal-weights state-averaged (SA) RASSCF calculation of 4 excited electronic states using an active space of 14 electrons in 14 orbitals (SA5-RASSCF(14/14)). The size of the Davidson Hamiltonian was 4 eigenpairs. The optimized pseudo-natural molecular orbitals (MOs) at the Franck-Condon point are shown in Figure S1. Of those, the $\pi(\mathrm{n} = 6)$ to $\pi(\mathrm{n} = 10)$ MOs were included in the full-CI, RAS2 expansion. RAS1 comprised of the remaining formally occupied, RAS3 the formally virtual orbitals. Excitations from RAS1 into RAS2 and RAS3, as well as excitations from RAS1 and RAS2 into RAS3 were included up to double excitations (notation 2:4/5:2). This treatment captures most of the solutions principal components (static correlation), capable of reproducing the full CASSCF(14/14) result with meV-accuracy at the Franck-Condon point.

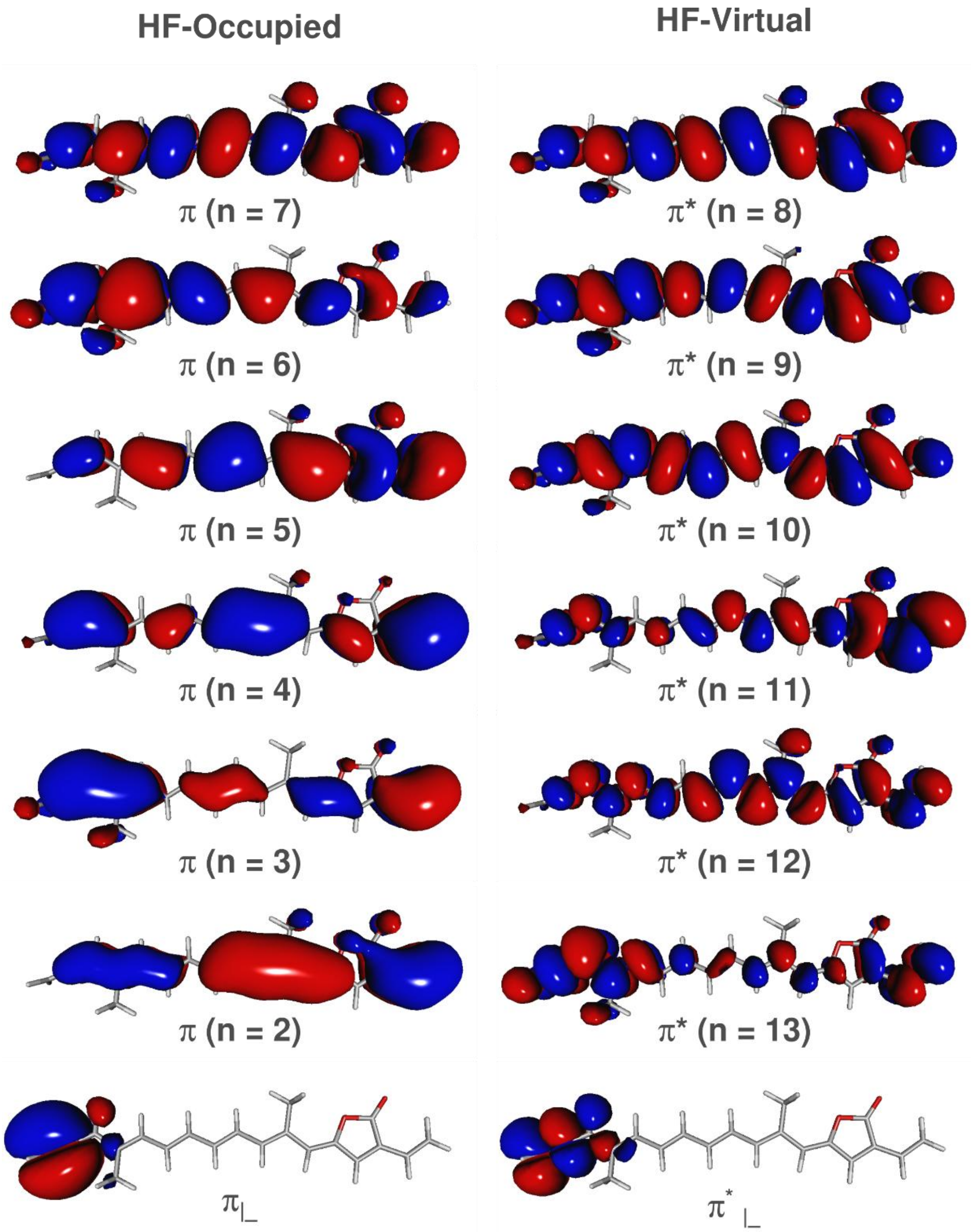

Figure S2. SA5-RASSCF(14/14, 2:4/5:2) pseudo-natural active orbitals (isovalue = 0.01) at the XMS-CASPT2-optimized Franck-Condon point, sorted by symmetry and occupancy of the HF determinant.

For the multi-state part of the perturbation theory calculation, the single-state single-reference (SS-SR) internal contraction scheme was used. Level-shifting of 0.3 eV was applied via the σ^2-regularisation.[4] The default IPEA shift was deactivated, motivated by recent results for small organic chromophores.[5] Multi-reference perturbation theories can be considered as the 'gold standard' for the characterization of excited states.[6] To correctly describe the electronic structure at (near-) degeneracies (see below SI Sec. **3.1**), quasi-degenerate perturbation theory

is required. We employed XMS-CASPT2 that showed smoother potential energy surfaces (PES), compared to multi-state (MS), extended dynamically weighed multi-state (XDW) and rotated multi-state (RMS) CASPT2 at or near conical intersections (CoIns).[7]

2.2 Principal Component Analysis and Relative Mean Bond Length (RBML) Metric Definition

A reaction coordinate describing the collective nuclear displacements in excited states connecting the Franck-Condon geometry and the $1\widetilde{B_u}$ and $2\widetilde{A_g}$ minima was generated by non-linear internal coordinate interpolation of the lowest three XMS-RASPT2 optimized minima. The collective displacement vector was obtained by principal component analysis (explained variance of 95 %), and discretized to 14 single-point evaluations.[8] The lowest PCA coordinate reflects the bond-order inversion of single and double bonds and the value of relative mean bond length (RBML) was computed as the ratio between N formal single bonds and M formal double bonds (cf. Figure S3):[9]

$$RBML = \frac{M \sum_i^N r(C-C)_i}{N \sum_j^M r(C=C)_j}$$

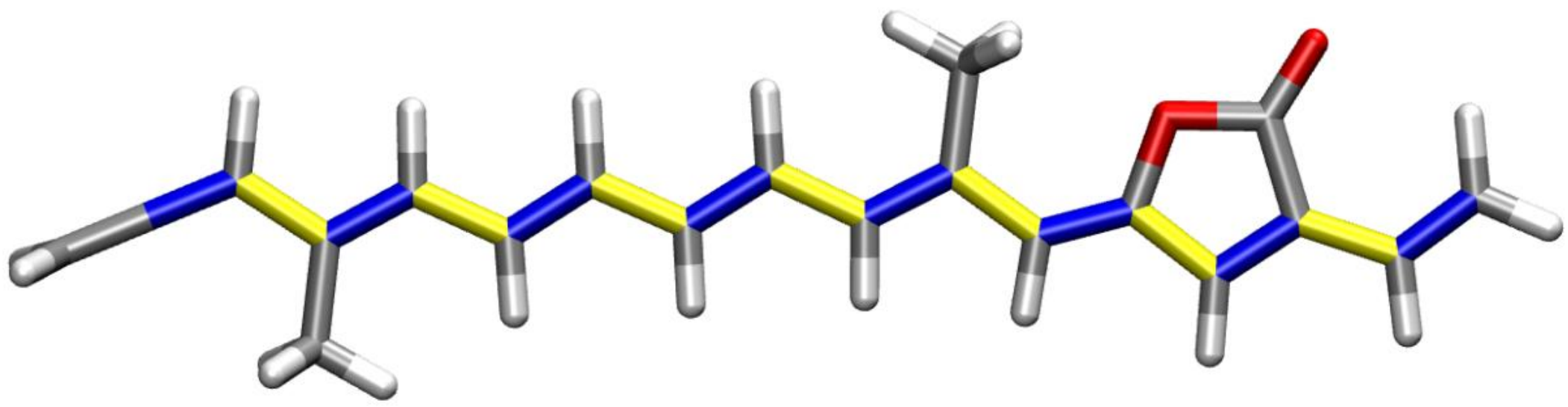

Figure S3. Geometry of the model system at the Franck-Condon point. For the calculation of the RMBL value, blue coloring signifies formal double bonds, yellow formal single bonds of the polyene chain.

2.3 Geometry Optimization

Geometry optimization was performed at the XMS(4)-RASPT2(14/14, 2:4/4:2)/cc-pVTZ level of theory. The deviation in CI expansion and basis set has a minor geometric effect on minimum geometries, both methods yield qualitatively identical surfaces and re-optimization was not performed. Optimized geometries primarily served as guess for the scan coordinate generation, generated via interpolation (see above)

The gas-phase S_1 minimum corresponds to the $2\widetilde{A_g}$, the S_2 minimum to the $1\widetilde{B_u}$ diabatic surface, respectively (cf. Figure S4). Both geometries were found to be reasonable approximations to the non-equilibrium, implicit-solvation geometries under an arbitrary dielectic constant (cf. main text).

3. Supporting Simulation Results

3.1 Character of the Lowest Excited States

Given that the treatment of explicit electronic correlation via the RASSCF method included 4 electrons (full-CI) and 14 electrons (RAS-CI) (i.e. small in comparison to the total number of electrons), the CI expansion coefficients are assessed to remain informative of the excited state character. Pseudo-symmetry labels were assigned by comparing to the MO node distribution of the corresponding symmetric polyene (all-trans-quadrodeca-1,3,5,7,9,11,13-heptaene). Amongst the lowest 3 eigenpairs, the 5 lead configuration state functions (CSFs) (4 pseudo-even, 1 pseudo-odd parity) are shown in Figure S4. The electronic ground state is dominated by the closed-shell HF configuration and acquires single excitation character upon bond order inversion. The mixing between pseudo-symmetries is possible due to asymmetric substitution. For the lowest two excited states, a state order inversion is evident as the even and odd excited CSFs exchange (RBML $\cong$ 1.03). Within the Franck-Condon region, the $1\widetilde{B_u}$ state is of single, the $2\widetilde{A_g}$ state of pronounced multi-refence character, congruent with literature findings.[9] In the strongly inverted regime (RBML < 1.0), the coefficients of all lead CSFs begin to equalize, signifying 3-state mixing in a diabatic picture, such that transition between the ground and first excited state becomes weakly symmetry-allowed.

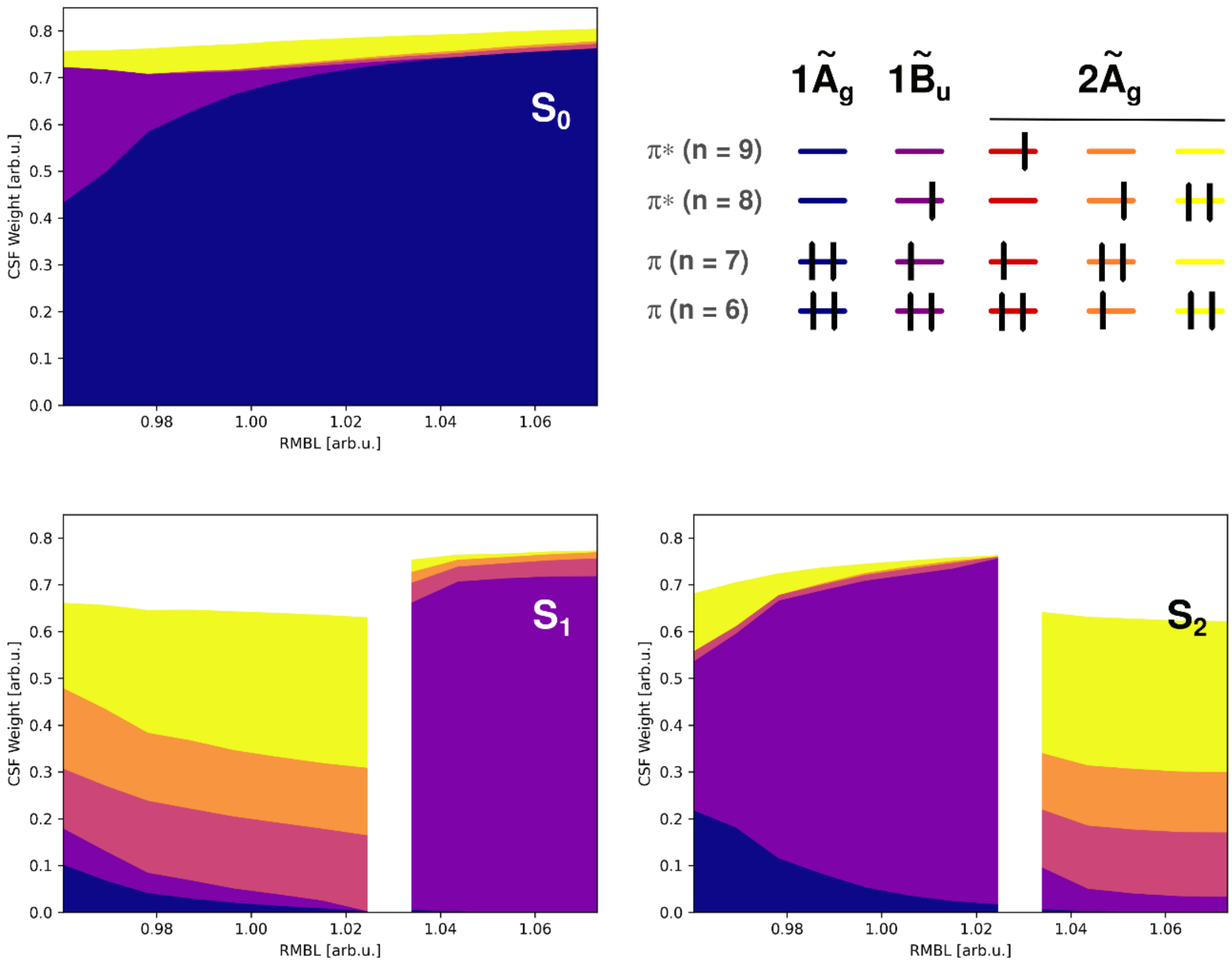

Figure S4: Lead CSF pseudo-symmetry assignment (top right) and coefficient distributions amongst the S_0, S_1 and S_2 adiabatic states along the scan coordinate (top left, bottom). MO labels are the same as in Figure S2. $1\widetilde{A_g}$, $1\widetilde{B_u}$, $2\widetilde{A_g}$ denote the pseudo-symmetry labels inherited from the symmetric polyene scaffold.

3.2 Gauge-Invariance of XMS-CASPT2 Oscillator Strengths

Since the XMS-CASPT2 method exhibits a slow basis set convergence, a comparison between length and velocity gauge representation of the oscillator strength is warranted. Table S1 demonstrates convergence with respect to the basis set size, since both representations yield a qualitatively identical picture along the interpolation coordinate.

Table S1: Comparison between length (len.) and velocity (vel.) gauge representation of the oscillator strengths between the ground state (S_0) and the first two excited states (S_1, S_2).

**RMBL [arb.u.]**	f_{osc}^{S0S1} **(len.) [arb.u.]**	f_{osc}^{S0S1} **(vel.) [arb.u.]**	**Difference [%]**	f_{osc}^{S0S2} **(len.) [arb.u.]**	f_{osc}^{S0S2} **(vel.) [arb.u.]**	**Difference [%]**
0.951	0.222	0.293	24.2	1.985	2.624	24.3
0.96	0.111	0.146	24.2	2.144	3.0	28.5
0.969	0.061	0.082	25.4	2.232	3.231	30.9
0.978	0.031	0.04	23.8	2.264	3.402	33.5
0.987	0.028	0.042	34.0	2.262	3.486	35.1
0.996	0.024	0.038	37.5	2.256	3.528	36.1
1.006	0.022	0.037	41.0	2.239	3.556	37.0
1.015	0.015	0.026	43.0	2.225	3.578	37.8
1.024	0.008	0.009	15.1	2.213	3.594	38.4
1.034	1.989	3.231	38.4	0.213	0.356	40.2
1.044	2.067	3.364	38.6	0.119	0.198	40.0
1.053	2.06	3.346	38.4	0.11	0.18	39.0
1.063	2.04	3.295	38.1	0.115	0.184	37.5
1.073	2.013	3.222	37.5	0.128	0.2	35.9

3.3 Comparison to DFT/MRCI

Besides XMS-CASPT2, we employed the semi-empirical, black-box method DFT/MRCI to validate our results.[10, 11, 12, 13] Along the interpolation coordinate, the adiabatic potentials and oscillator strength distribution were qualitatively recovered (cf. figure S5). The avoided crossing between S1 and S2 appeared more pronounced and systematic red shifts were noted, ranging from 0.1-0.2 eV for the $2\widetilde{A_g}$ state and 0.2-0.35 eV for the $1\widetilde{B_u}$ state 0.2-0.35 eV. Thus enhanced mixing between the diabates is reflected in a greater brightness of the S_0S_1 transition in the inverted regime (RMBL < 1), which gained 10% in relative oscillator strength compared to XMS-CASPT2.

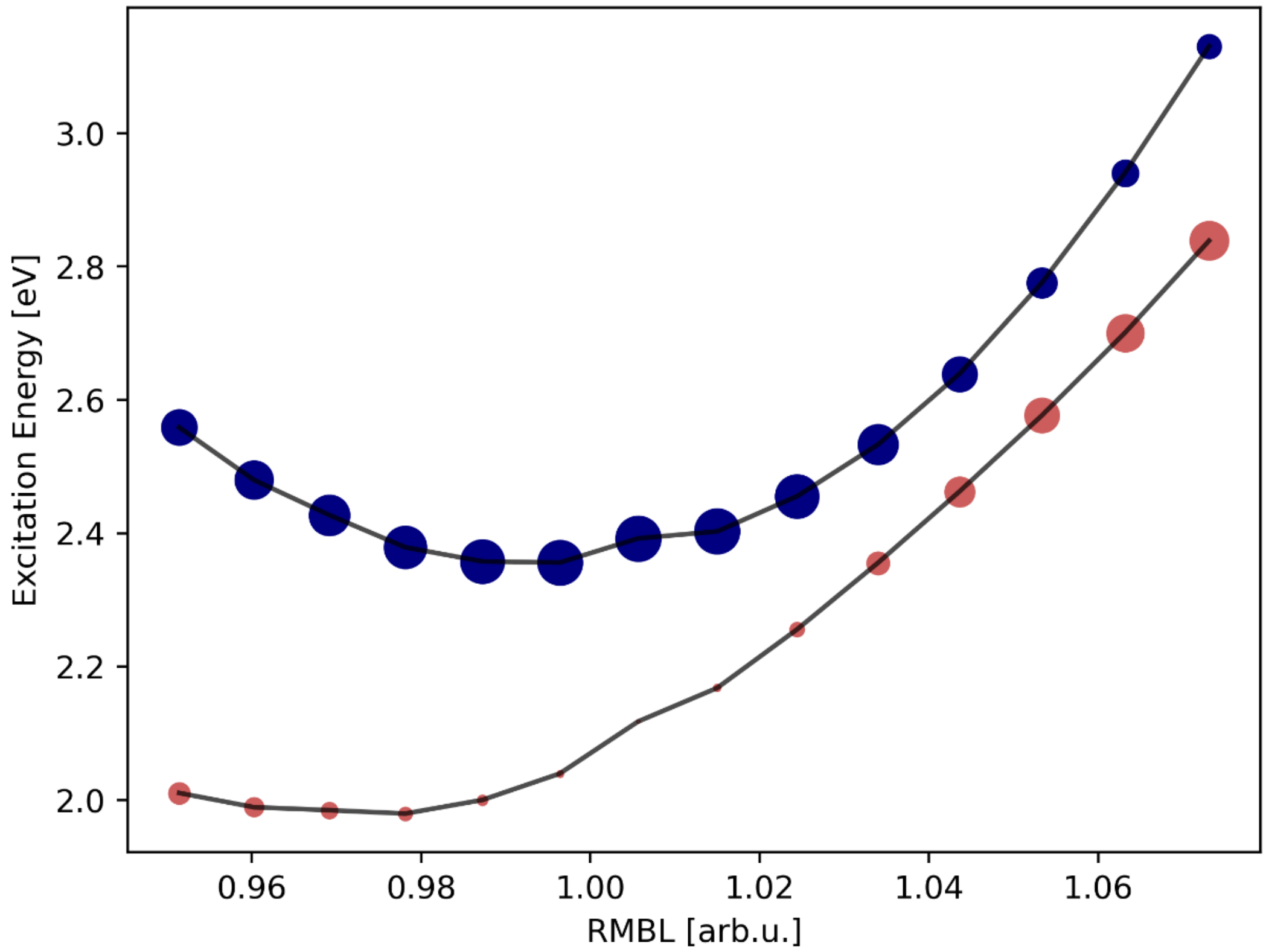

Figure S5: DFT/MRCI potentials (lines) and oscillator strengths (dots) along the interpolation coordinate. Oscillator strengths of the S_1 (red) and S_2 (blue) are renormalized to the maximum value of the dataset.

4. Computational Results

The geometries of optimized stationary points on the potential energy surfaces (PES), as well as a Molcas RasOrb file of the SA5-RASSCF(14/14, 2:4/5:2) active space are provided within a separate repository [OpenLMU].

5. Sketch of potential energy surfaces before and after solvent relaxation

As evident from Stark spectroscopy,[14] the bright $1B_u$ state possesses a significantly higher dipole moment than the $1A_g$ and $2A_g$ states. Upon excitation into the $1B_u$ state, solvent molecules will respond to the increased dipole moment and eventually align with the solute dipole moment, thus selectively stabilizing the $1B_u$ diabatic state. In a continuum description of the solvent, the solvation process is captured by the counter alignment of the cavity field of the solvent with the dipole moment of the solute. In first order, the magnitude of the cavity field is expected to be proportional to the dielectric constant of the solvent, and it's time-evolution

to follow the solvent response function. In polar solvents, such as ethylene glycol, this solvent stabilization of the diabatic $1B_u$ state may suffice to eventually invert the excited state ordering of $2A_g$ and $1B_u$ states, as shown in Fig. S6. In this picture, the commonly inferred 'ICT state' is then associated with the solvent-adapted $1B_u$ state. Research validating this working hypothesis within a continuum solvation model is currently pursued in our laboratory.

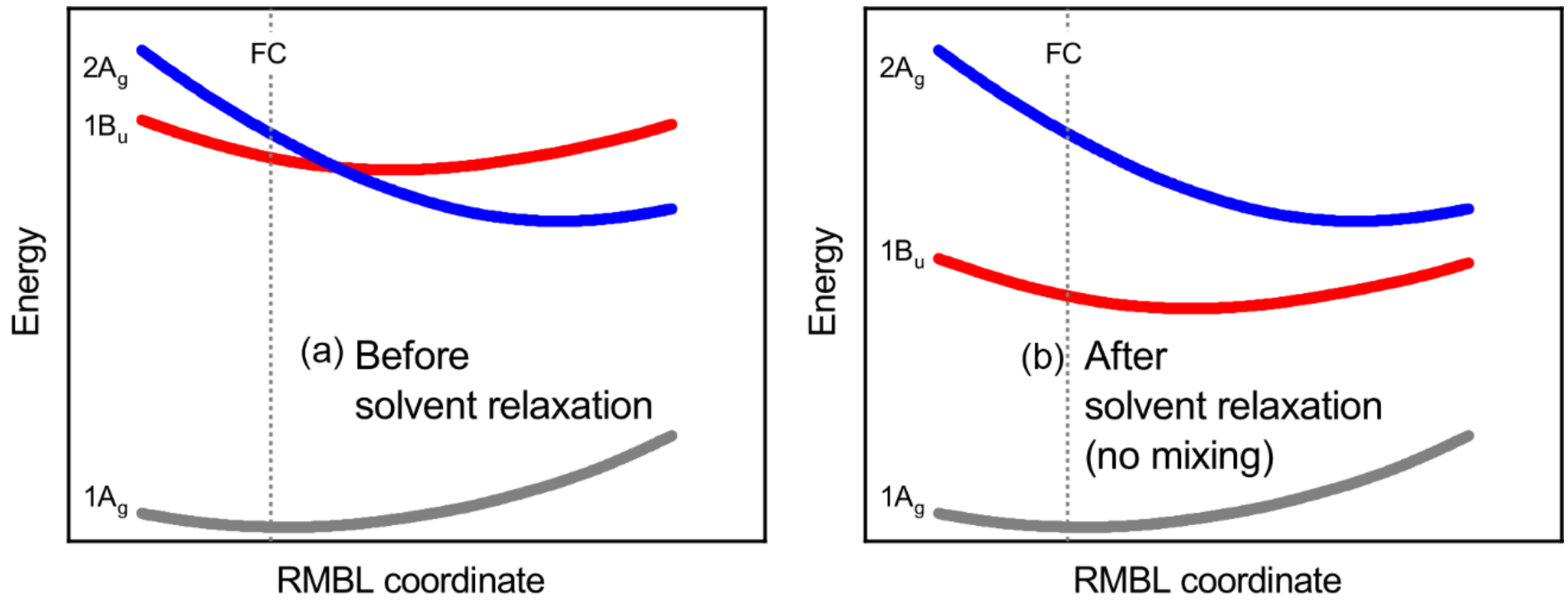

Figure S6. Sketch of the potential energy surfaces in peridinin before and after solvent relaxation. (a) is equivalent to Fig. 3 in the main text. (b) qualitative sketch of the situation after solvent relaxation, with increased stabilization of the $1B_u$ surface.

References

(1) Li Manni, G; Galván, I. F.; Alavi, A.; Aleotti, F.; Aquilante, F.; Autschbach, J.; Avagliano, D.; Baiardi, A.; Bao, J. J.; Battaglia, S.; Birnoschi, L.; Blanco-González, A.; Bokarev, S. I.; Broer, R.; Cacciari, R.; Calio, P. B.; Carlson, R. K.; Couto, R. C.; Cerdán, L.; Chibotaru, L. F.; Chilton, N. F.; Church, J. C.; Conti, I.; Coriani, S.; Cuéllar-Zuquin, J.; Daoud, R. E.; Dattani, N.; Decleva, P.; de Graaf, C.; Delcey, M. G.; De Vico, L.; Dobrautz, W.; Dong, S. S.; Feng, R.; Ferré, N.; Filatov, M.; Gagliardi, L.; Garavelli, M.; González, L.; Guan, Y.; Guo, M.; Hennefarth, M. R.; Hermes, M. R.; Hoyer, C. E.; Huix-Rotllant, M.; Jaiswal, V. K.; Kaiser, A.; Kaliakin, D. S.; Khamesian, M.; King, D. S.; Kochetov, V.; Krośnicki, M.; Kumaar, A. A.; Larsson, E. D.; Lehtola, S.; Lepetit, M.-B.; Lischka, H.; Ríos, P. L.; Lundberg, M.; Ma, D.; Mai, S.; Marquetand, P.; Merritt, I. C. D.; Montorsi, F.; Mörchen, M.; Nenov, A.; Nguyen, V. H. A.; Nishimoto, Y.; Oakley, M. S.; Olivucci, M.; Oppel, M.; Padula, D.; Pandharkar, R.; Phung, Q. M.; Plasser, F.; Raggi, G.; Rebolini, E.; Reiher, M.; Rivalta, I.; Roca-Sanjuán, D.; Romig, T.; Safari, A. A.; Sánchez-Mansilla, A.; Sand, A. M.; Schapiro, I.; Scott, T. R.; Segarra-Martí, J.; Segatta, F.; Sergentu, D.-C.; Sharma, P.; Shepard, R.; Shu, Y.; Staab, J. K.;

Straatsma, T. P.; Sørensen, L. K.; Tenorio, B. N. C.; Truhlar, D. G.; Ungur, L.; Vacher, M.; Veryazov, V.; Voß, T. A.; Weser, O.; Wu, D.; Yang, X.; Yarkony, D.; Zhou, C.; Zobel, J. P.; Lindh R. The OpenMolcas *Web*: A Community-Driven Approach to Advancing Computational Chemistry. *Journal of Chemical Theory and Computation* **2023**, 19(20), 6933–6991.

(2) Taffet. E. J; Scholes, G. D., Peridinine Torsional Distortion and Bond-Length Alternation Introduce Intramolecular Charge-Transfer and Correlated Triplet Pair Intermediate Excited States, *The Journal of Physical Chemistry B* **2018**, 122(22), 5835-5844.

(3) Wagner, Nicole L.; Greco, Jordan A.; Enriquez, Miriam M.; Frank, Harry A.; Birge, Robert R. The Nature of the Intramolecular Charge Transfer State in Peridinin. *Biophysics Journal* **2013**, *104* (6), 1314-1325.

(4) Battaglia S.; Fransén L.; Galván, I. F.; Lindh, R. Regularized CASPT2: an intruder-state-free approach. *Journal of Chemical Theory and Computation **2022**,* 18(8), 4814–4825.

(5) Zobel, J.P; Nogueira, J.J.; González, L. The IPEA Dilemma in CASPT2. *Chemical Science* **2017**, *8,* 1482-1499.

(6) Chattopadhyay, S.; Chaudhuri, R. K.; Mahapatra, U. S.; Ghosh, A.; Ray, S. S. State-Specific Multireference Perturbation Theory: Development and Present Status. *WIREs Computational Molecular Science* **2016**, *6,* 266-291.

(7) Nishimoto, Y.; Battaglia, S.; Lindh, R. Analytic First-Order Derivatives of (X)MS, XDW, and RMS Variants of the CASPT2 and RASPT2 Methods. *Journal of Chemical Theory and Computation* **2022**, *18,* 4269-4281.

(8) Pointner, F. L.; Reiter, S.; Fingerhut, B. P.; de Vivie-Riedle, R., MonicaMD: Molecules and Internal Cluster Analysis of Molecular Dynamics Simulations. *Journal of Chemical Information and Modeling* **2026**, 66(10), 5940-5952.

(9) J. Zhang, C. Jaschke, B. P. Fingerhut, T. Elsaesser, Transient Electronic Polarizability of β-Carotene from Ultrafast Terahertz Stark Spectroscopy, *Journal of Physical Chemistry Lett*ers 2026, 17, 1613–1620.

(10) Grimme, S.; Waletzke, M. A combination of Kohn–Sham density functional theory and multi-reference configuration interaction methods. *The Journal of Chemical Physics* **1999**, *111* (13), 5645-5655.

(11) Kleinschmidt, M.; Marian, C. M.; Waletzke, M.; Grimme, S. Parallel multireference configuration interaction calculations on mini-beta-carotenes and beta-carotene. *J. Chem. Phys.* **2009**, *130* (4), 044708.

(12) Dombrowski, D. R.; Schulz, T.; Kleinschmidt, M.; Marian, C. M. R2022: A DFT/MRCI Ansatz with Improved Performance for Double Excitations. *The Journal of Physical Chemistry A* **2023**, *127* (8), 2011-2025.

(13) Neese, F. The ORCA program system. *Wiley Interdisciplinary Reviews: Computational Molecular Science* **2012**, *2*.
